# Features of Structure and Properties of pHEMA-gr-PVP Block Copolymers, Obtained in the Presence of Fe^2+^

**DOI:** 10.3390/ma13204580

**Published:** 2020-10-14

**Authors:** Oleksandr Grytsenko, Ludmila Dulebova, Oleh Suberlyak, Volodymyr Skorokhoda, Emil Spišák, Ivan Gajdoš

**Affiliations:** 1 Department of Chemical Technology of Plastics Processing, Lviv Polytechnic National University, 12 St. Bandera, 79013 Lviv, Ukraine; oleksandr.m.grytsenko@lpnu.ua (O.G.); oleh.v.suberliak@lpnu.ua (O.S.); volodymyr.y.skorokhoda@lpnu.ua (V.S.); 2 Department of Technologies, Materials and Computer Aided Production, Technical University of Košice, 74 Mäsiarska, 04001 Košice, Slovakia; emil.spisak@tuke.sk (E.S.); ivan.gajdos@tuke.sk (I.G.)

**Keywords:** polyvinylpyrrolidone, 2-hydroxyethylmethacrylate, hydrogel, block polymerization, iron sulfate, graft copolymer, crosslinked polymer, polymer network

## Abstract

This paper presents the research results of the copolymer structure and properties of 2-hydroxyethylmethacrylate (HEMA) with polyvinylpyrrolidone (PVP) and their hydrogels, obtained by block polymerization in the presence of iron sulfate (II). By the methods of chemical analysis, IR spectroscopy, Thermogravimetric (TG) and Differential Thermal Analysis (DTA), the course of grafted copolymerization of HEMA on PVP with the formation of a cross-linked copolymer was confirmed. The results received by scanning electron microscopy showed that due to the copolymerization of HEMA with PVP, macroporous hydrogels with a pore size of 10–30 μm were obtained. The peculiarities of the structure formation of the obtained copolymers depending on the initial composition formulation were established and their structural parameters were investigated: PVP grafting efficiency, PVP content in copolymer, molecular weight of internodal fragment of polymer network, crosslinking degree, and crosslinking density. The interrelation of sorption–diffusion, physical–mechanical and thermophysical properties along with the structure of the obtained materials was proved. It was shown that with the increasing PVP content in the original composition, the efficiency of its grafting and crosslinking density of the polymer network decreased, but the surface hardness, heat resistance, sorption capacity of copolymers in the dry state, as well as ion permeability and elasticity in the swollen state increased, while their tensile strength deteriorated. It is proved that by changing the original composition formulation it is possible to change the structure and hence the properties of the copolymers in the desired direction.

## 1. Introduction

In recent years, the synthesis and study of polymer hydrogel materials is one of the priority areas of polymer science and practice, which is actively developing [1,2,3,4,5,6].

Hydrogels were obtained by water saturation of highly hydrophilic structured polymers, usually during the swelling of which there is a physical transition from a vitreous to a highly elastic state. Due to the sorption of water or hydroxyl-containing organic solvent in such cross-linked hydrophilic polymers, a two-phase system is formed, which consists of polymer chains, chemically and (or) physically interconnected, and water, which fills the free space in the network [2,7,8]. Hydrogels are characterized by a high sorption capacity for low molecular weight compounds and permeability for liquids and gases, which is a prerequisite for their use in various fields of science and technology [3,4,7,9]. Those materials have acquired special practical application in medicine and biotechnology [9,10,11,12,13]. Due to their structure, which resembles the structure of living tissues, hydrogels are characterized by good biocompatibility, which allows them to be used in direct contact with a living organism [14,15,16].

The performance properties of hydrogel materials depend on the nature of the components of the original composition [17,18]. Besides, the technological obtaining features of hydrogel material do not play the final role in the selection of such types of material for the purpose of manufacturing products for specific practical use [3]. The main influence on these features accomplish the type of initiation system [19].

Among a wide range of hydrophilic polymers, the copolymers based on 2-hydroxyethylmethacrylate (HEMA) with polyvinylpyrrolidone (PVP) are promising [20,21,22,23,24,25,26,27,28,29,30,31,32]. These materials are characterized by technological advantages of obtaining and a combination of unique properties, which open up the prospect of their use, such as hydrogel medical dressings [21,22], contact lenses [23,24], vascular prostheses [25], materials for regeneration of injured tissues [26], hydrogel membranes [27], enzyme immobilization systems [28], drug delivery systems [29,30], for the removal of adhesives from the back of canvas paintings [31], electrically controlled elements of optical systems [32], etc.

By previous studies we developed compositions based on PVP, mono and dimethacrylic esters of glycols, which polymerize under the action of metal ions of variable oxidation states [33,34]. The use of the PVP/Me^n+^ complex provides the obtaining of copolymers of HEMA with PVP (pHEMA-gr-PVP) at a high rate, at room temperature, in the light, in the air, and in one stage. Depending on the composition formulation, content, nature of the solvent and the concentration of the metal salt, the duration of formation (with a limiting yield of a polymer 98–99%) of such copolymers can be varied within wide limits: from 15 to 150 min. It was found that the most effective towards polymerization rates among the investigated metal salts is iron (II) sulfate [33]. Polymerization of HEMA in the presence of PVP under the action of Fe^2+^ occurs with high rates at room temperature, in air, which makes it possible to significantly simplify and lower the cost of the process, reduce its duration and expand the possibilities of its use.

The main component of the hydrogel network was 2-hydroxyethylmethacrylate, chosen due to its unique properties. Because HEMA can be easily polymerized, possesses a hydrophilic pendant group, and can form hydrogels, an increasing number of applications have been found in various biomedical fields [35]. The growing interest in polymer hydrogels based on HEMA is primarily due to the fact that they exhibit excellent biocompatibility, blood compatibility, cytotoxicity, cell compatibility, and low thrombogenicity [36,37]. In addition, hydrogels based on HEMA have better mechanical properties, compared to hydrogels with a different nature [38].

The method of grafted copolymerisation of HEMA on PVP appears to be particularly promising with significant possibilities of hydrogel polymeric matrix formation [39]. PVP is used by itself as a plasma substitute, sorbent, thickener of cosmetic ointments, a soluble drug carrier, a modifier for enzymes, and a comonomer in UV curable bioadhesives. PVP is also a very good choice for making hydrogels [40,41,42]. The use of PVP opens up additional opportunities for the synthesis and stabilization of metal nanopowders [43,44], modification of various substances and materials [45,46,47,48,49,50], the improvement of modern technologies [51,52,53], the obtainment of new functional materials [54,55,56,57] and, accordingly, the expansion their application field.

During the synthesis of copolymers pHEMA-gr-PVP, their structure and, consequently, properties depends on the content of PVP in the initial mixture. In turn, PVP is known [39] to be partially bound during polymerization and partially washed away during hydration. The properties of the polymer hydrogel, as any other material, depend on its structure. It is known that from the same polymer it is possible to make materials with various properties only due to a change of its structural parameters. Therefore, the study of the structure of copolymers and hydrogel materials based on them, as well as the study of the interrelation of structure with properties, has important scientific and practical value for the targeted production of materials with specified characteristics.

The aim of this work was to experimentally confirm the formation of grafted crosslinked copolymers pHEMA-gr-PVP obtained in the presence of Fe^2+^ ions, and to establish the interrelation of properties between the synthesized copolymers and their structure depending on the initial composition formulation.

## 2. Materials and Methods

### 2.1. Materials

The following substances were used: 2-hydroxyethylmethacrylate (Sigma Chemical Co., Saint Louis, MO, USA), which was purified and distilled in a vacuum (residual pressure = 130 N/m^2^, T_B_ = 351 K); polyvinylpyrrolidone (AppliChem GmbH, Darmstadt, Germany) of high purity with MM 28,000 was dried at 338 K in a vacuum for 2–3 h before use; iron (II) sulfate was used of p.a. grades.

The amount of PVP in the initial composition was used in range of 10 ÷ 40 mass parts. The lower limit of the PVP content is caused by the fact that with less content of it, the hardening time of the compositions significantly increases (up to 24 h). The upper limit is caused by technological complications: with higher PVP content, the duration of its dissolution in HEMA increases, and the viscosity of the composition, which is difficult to dispense and deaerate, increases as well.

### 2.2. Synthesis Technique of pHEMA-gr-PVP Copolymers

Copolymerization of HEMA with PVP was carried out in the block with the presence of iron (II) sulfate at room temperature for 0.3–1.5 h (depending on the composition formulation). The calculated amount of PVP and FeSO_4_ were weighed according to the given formulation, HEMA was dosed by volume method. Into 1/3 part of the HEMA amount, required for polymerization, a certain amount of FeSO_4_ (0.05 wt.%) was dissolved. The required amount of PVP was dissolved in the rest of the HEMA content. Stirring was performed at room temperature until complete dissolution of FeSO_4_ and PVP in HEMA. Solutions of FeSO_4_ and PVP in HEMA were mixed to obtain a homogeneous mixture, without insoluble agglomerates and mechanical inclusions, which was processed by casting method into a polymerization mold. The obtained products were rinsed in distilled water until unreacted HEMA and PVP was completely removed.

### 2.3. Measurements and Characterization

#### 2.3.1. Standard Methods of Instrumental Research

By using a PARAGON 1000 FTIR spectrometer, equipped with a single-horizontal Golden Gate ATR cell (Perkin-Elmer, Waltham, MA, USA), the attenuated total reflectance Fourier transform infrared (ATR FTIR) spectra were taken. The form of the used samples was a pellet. The spectra were taken after 16 scans, at a resolution of 4 cm^−1^, in the range of 500 to 4000 cm^−1^. Differential thermal (DTA) and thermogravimetric analysis (TG) were performed by using Paulik–Paulik–Erdey derivatograph. For the research samples weighing 0.1 g in powdered form were used. The experiment was performed in air, at a heating rate of 10 °C/min at a temperature within 0 ÷ 700 °C. Studies of the structure of the copolymers were carried out by scanning electron microscopy method (SEM), using scanning electron microscope-microanalyzer PEMMA-102-02 (JSC “SELMI”, Sumy, Ukraine). The research was carried out under the following conditions: range of magnification change within 10 ÷ 30 × 10^4^, range of accelerating voltage change within 0.2 ÷ 40 keV, resolution up to 5.0 nm.

#### 2.3.2. PVP Grafting

The method of determining PVP is based on the formation of its colored complex with iodine [58]. To determine the PVP that entered into the grafting reaction with the monomer, the amount of unbounded PVP in the copolymer was found by studying its water extracts. The research was performed on a photocolorimeter KFK-2MP (JSC, Zagorskiy Optiko-Mekhanicheskiy Zavod, Sergijev Posad, Russia), with colored filter of λ = 590 nm.

With the knowledge of the PVP content in the original composition and extract, its efficiency (*f*, %) and the degree of grafting (*p*, %) were calculated:

(1) $$f=\frac{m_{1}}{m}\cdot 100\%,$$

(2) $$p=\frac{m_{1}}{M}\cdot 100\%,$$

where *m*_1_–the amount of grafted PVP, g; *m*–the PVP content in the original composition, g; *M*–the total mass of the copolymer, g.

#### 2.3.3. The Molecular Weight between Cross-Links in the Polymer Network

The molecular weight between cross-links in the polymer network (*M_C_*, kg/mol) was determined by the equilibrium modulus of high elasticity *E*, using the formula [59]:

(3) $$E=3\frac{\rho RT}{M_{C}},$$

where *E*–equilibrium modulus of high elasticity, N/m^2^; *ρ*–polymer density, kg/m^3^; *R*–gas constant, J/(mol∙K); *T*–test temperature, K; *M_C_*–molecular weight between cross-links in the polymer network, kg/mol.

For the studies cylindrical samples of 8 mm high and ∅ 15 mm, hydrated to equilibrium state and extracted until final removal of unbound PVP were used. These samples were affected to different loads to constant deformations, in which the sample was deformed less than 30%. Measurements were performed on a Hepler consistometer.

On the basis of the obtained data, the dependence of the equilibrium high elasticity deformation ε (%) on the compression stress σ (N/m^2^) was plotted. The equilibrium modulus of high elasticity *E* was found by the angle *α* of the slope of line σ_i_ = f (ε_i_):

(4) E=tgα,

According to Formula (3), the *M_C_* was determined. By using *M_C_*, the values of the degree of crosslinking (5) and the crosslinking density (6) of the polymer network were calculated:

(5) $$\nu =\frac{1}{M_{C}},$$

(6) $$\mu =\frac{\rho }{2M_{C}},$$

#### 2.3.4. Properties of Copolymers

Boundary water absorption (*W*, wt.%) was determined by the weight method as the difference between dry (*m*_0_, g) and swollen (*m*_1_, g) samples:

(7) $$W=\frac{m_{1}-m_{0}}{m_{1}}\cdot 100\%,$$

Hydrogel swelling factor was determined as a ratio of sizes of dry (*d_d_*, mm) and swollen (*d_s_*, mm) samples:

(8) $$k=\frac{d_{s}}{d_{d}},$$

The kinetics of swelling were characterized by the degree of water absorption *α*, g (H_2_O)/g (polymer). *α* at time t was determined by the ratio of the mass of absorbed liquid by the polymer during swelling (*m_t_*) to the mass of dry polymer (*m*_0_):

(9) $$\alpha_{t}=\left(m_{t}-m_{0}\right)/m_{0},$$

Samples of 1.3 ± 0.1 mm thick were used for the studies.

The ion permeability of hydrogels was evaluated by the ion permeability coefficient (*K_NaCl_*, mol/m^2^⋅s) and the diffusion coefficient (*K_D_*, m^2^/s). The studies were performed in a two-chamber cell according to the method of determining the amount of model salt (NaCl) diffused from one chamber through a sample of hydrogel, based on the measurement of the electrical conductivity of the solution in the second chamber. Measurements of the electrical conductivity of the solution were performed by using a rheochord bridge. The device was pre-calibrated with aqueous solutions of known NaCl concentrations. *K_NaCl_* and *K_D_* were determined by the Formulas:

(10) $$K_{NaCl}=\frac{G}{\tau \cdot C\cdot S},$$

(11) $$K_{D}=\frac{l^{2}}{6\cdot \tau_{d}},$$

where: *G*—the amount of salt that penetrated through the polymer sample, mol; *S*—the effective area of the sample surface, m^2^; *C*—the concentration of the original salt solution, wt.%; *τ*—the duration of the process, s; *l*—the thickness of the sample, m; *τ_d_*—the delay time, s.

The surface hardness of dry samples (with diameters Ø 12 ± 0.1 mm and height h = 5 ± 0.01 mm) was found according to the conic yield point in a Höppler consistometer at 293 °K by pressing a steel cone with a vertex angle of 58°08′ into a polymeric specimen under a load of 5.0 kg for 60 s [60].

Determination of heat resistance of copolymers was carried out according to the method ISO 306:2013 «Plastics-Thermoplastic materials-Determination of Vicat softening temperature», which consists of determining the temperature at which a standard indenter with a flat bottom surface (Ø 1.128 ± 0.008 mm) is pressed under the action of the load to a depth of 1 mm in the test sample, which is heated at a constant speed. Studies were performed by using a Höppler consistometer.

Tensile strength at break (*σ_br_*, Pa) and elongation at break (*ε_br_*, %) of equilibrium swollen hydrogel films were determined by using a tensile testing machine “Kimura” 050/RT-601U (Kimura Machinery, Hiroshima, Japan) with the force up to 10 kN. While measuring the width and thickness of the film samples in three places, the cross section of the samples was determined. During the study, the load and elongation of the sample were measured: continuously, at the time of reaching the yield strength, and at the time of rupture of the sample. Tensile strength at break and elongation at break were determined by the formulas:

(12) $$\sigma_{br}=\frac{F_{br}}{A_{0}},$$

(13) $$\varepsilon_{br}=\frac{\Delta l_{br}}{l_{0}}\cdot 100\%,$$

where *F_br_*—the load during which the sample is breaking, N; *A*_0_—the initial cross section of the sample, mm^2^; Δ*l_br_*—a change in the estimated length of the sample at the moment of break, mm; *l*_0_—the initial estimated length of the sample, mm.

## 3. Results and Discussion

### 3.1. Study of the Structure of pHEMA-gr-PVP Copolymers

**Scheme 1:**
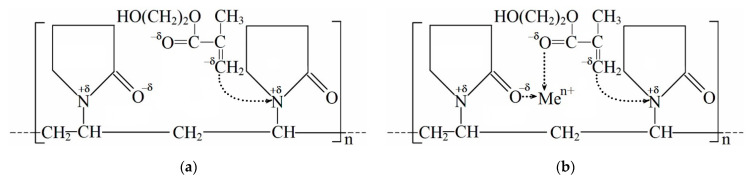
Complexes with charge transfer: 2-hydroxyethylmethacrylate (HEMA)/polyvinylpyrrolidone (PVP) (**a**) and HEMA/Me^n+^/PVP (**b**).

It was found that the high reactivity of HEMA/PVP compositions was caused by a so-called “matrix effect” [39], when monomer molecules are solvated on a polymer matrix (Scheme 1a), which increases the rate of polymerization. In this case, PVP plays the role of the matrix. In the direction of the polymer chains of PVP, the spatial orientation of HEMA occurs, which provides favorable kinetic growth conditions.

When adding metal salts with variable oxidation state to HEMA/PVP composition, the π-complex with participation of PVP, HEMA and Me^n+^ is formed. (Scheme 1b) [33,60]. The interaction occurs in the coordination sphere of the metal ion, which is accompanied by a significant weakening of the π-bond in the monomer.

**Scheme 2:**
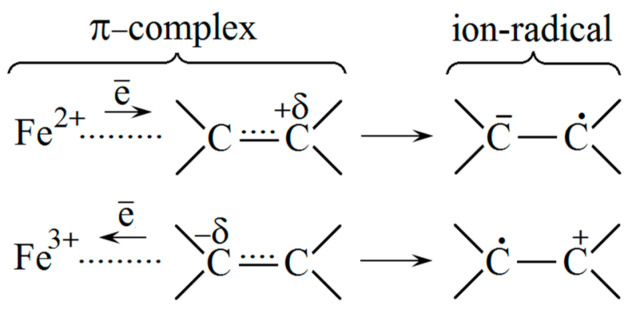
Possible options for initiating the polymerization by iron ions [33].

As a result of this interaction, Fe^2+^ gives one electron to the monomer molecule (resulting in the rupture of the double bond) with the formation of Fe^3+^ and the anion-radical of the monomer. The formed anion-radical can initiate further chain growth by both ionic and radical mechanisms. In the presence of Fe^3+^, obviously an electron transfer is carrying out from the monomer to Fe^3+^ and the formation of a cation-radical. Subsequently, the reaction proceeds by analogy with Fe^2+^. Thus, a constant restoration of the catalytic ability of the salt occurs, as shown in Scheme 2. In previous works [33], the authors found that the copolymerization of HEMA with PVP in the presence of ions of metals with variable oxidation state may occur by an ion-radical mechanism.

As a result, the copolymerization of HEMA with PVP takes place at a high rate and “limiting” yield of the polymer at room temperature.

It should be noted that without PVP in the experimental conditions, the polymerization of HEMA does not occur. With the introduction of even a small amount (1–2%) of PVP in the composition based on HEMA, the polymerization rate increases sharply, while almost no induction effect is observed.

The research results of the copolymerization kinetics of HEMA with PVP in the presence of metal ions [33] allow the prediction of the course of the reaction with the kinetic chain transfer on the PVP macromolecule and the grafted crosslinked copolymer formation.

**Figure 1:**
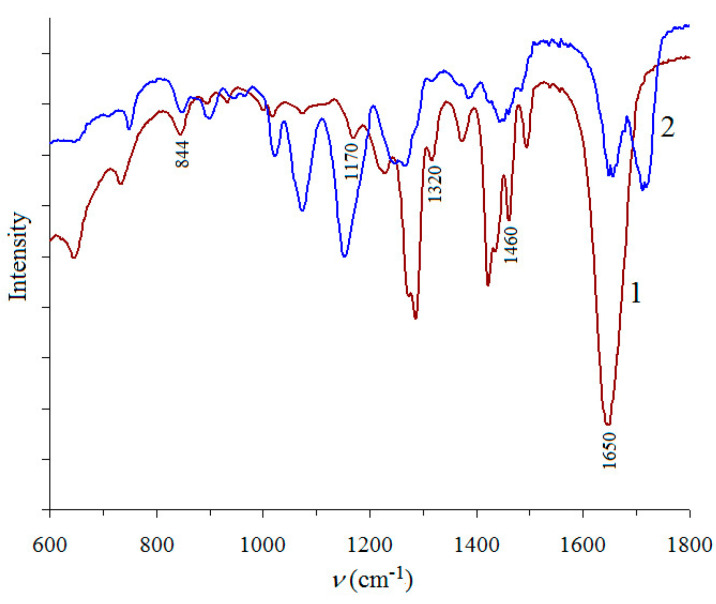
ATR FTIR spectra of PVP (**1**) and pHEMA-gr-PVP copolymer (**2**).

To confirm the formation of the grafted copolymer, IR spectra of PVP and pHEMA-gr-PVP copolymer was extracted by water until complete removal of unreacted PVP was obtained (Figure 1). Analysis of the obtained spectra showed that the characteristic bands of PVP in the ranges 650 cm^−1^, 844 cm^−1^, 1170 cm^−1^, 1320 cm^−1^, 1460 cm^−1^, 1650 cm^−1^ were presented in the spectrum of copolymer [34].

**Scheme 3:**
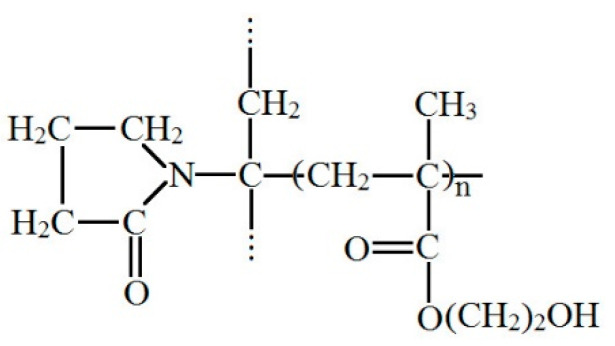
The structure of pHEMA-gr-PVP copolymer.

At the same time, the intensity of the band in the range of 1320 cm^−1^, which is related to the deformation vibrations of –CH group of the PVP carbochain, is significantly lower for water-extracted after polymerization of the copolymer, compared to the original PVP taken in the commensurate amount with its content in the copolymer. This fact gives the possibility to predict the grafting of HEMA to PVP with the participation of this group (Scheme 3).

In addition, the IR spectrum of the copolymer showed a significant decrease in the intensity of the characteristic PVP band in the range of 1372 cm^−1^, which indicates wagging vibrations of the CH_2_–CH bond of polyvinylpyrrolidone, and proves the participation of tertiary carbon of PVP in the grafting reaction [60].

**Figure 2:**
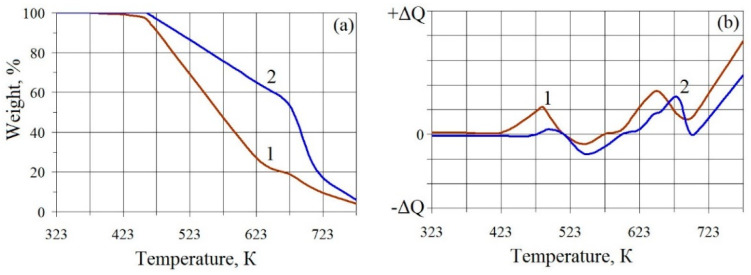
Thermogravimetric (**a**) and differential-thermal (**b**) analysis of (co)polymers: **1**–pHEMA/PVP mixture (pHEMA:PVP = 80:20 mass parts); **2**–pHEMA-gr-PVP copolymer (HEMA:PVP = 80:20 mass parts) [34].

Performed thermogravimetric (TG) and differential thermal (DTA) analyses of the copolymer with the ratio of HEMA:PVP = 80:20 and a mixture of polyHEMA: PVP with the same composition confirmed the formation of grafted copolymers HEMA with PVP (Figure 2).

The analysis of the TG curve of the homopolymer mixture indicates that the weight loss of the sample begins at about 423 K, while the copolymer begins to lose weight at approximately 463 K. On the DTA curve for the mixture, there is a peak of exothermic effect at 423–513 K, which by increasing temperature is replaced by endoeffect at 513–573 K, and in the temperature range 598–693 K, exothermic effect occurs again. Heat evolution at 423 K may be explained by thermal oxidation of weakly-bounded groups to the macromolecule, which is accompanied by further destruction with the evolution of volatiles. The endothermic effect is connected with heat, which is spent on the partial softening of the polymer which at 593 K starts to destruct. Thermal oxidation of the polymer at 423–513 K occurs on a place of tertiary hydrogens of PVP, which have the weakest connection with macromolecular chains, due to the electron shift to a nitrogen atom (Scheme 1). On the DTA curve of the copolymer, the exoeffect in the temperature range 423–513 K is almost absent, which indicates that the copolymer oxidizes slower than the mixture. However, the endoeffect in the temperature range 513–598 K and the exoeffect at 633–723 K are observed. The shift of the interval of the exothermic effect on the DTA curve to the region of high temperatures is explained by its greater thermal stability. This can be explained by the smaller number of tertiary hydrogen atoms in the PVP macromolecules, which are the part of the grafted copolymer, compared to their number in the PVP mixture.

Thus, TG and DTA investigations in combination with IR spectrometry data indicate the interaction of HEMA with PVP and the formation of grafted copolymer.

**Figure 3:**
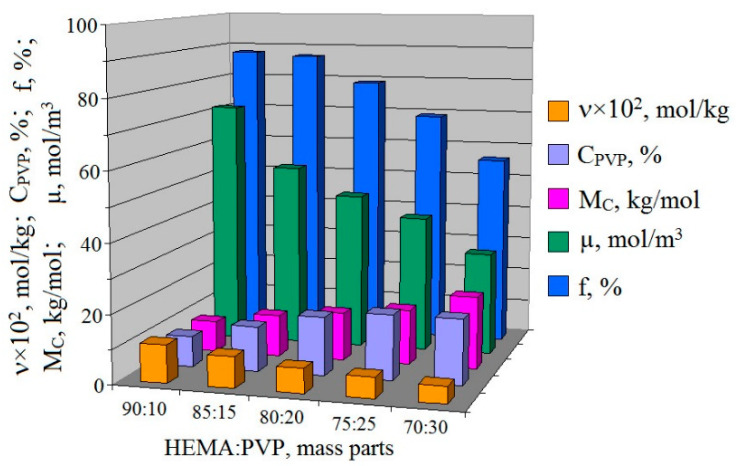
The effect of the initial composition formulation on the structural characteristics of pHEMA-gr-PVP copolymers.

However, it is known [34,39] that not the whole PVP enters the grafting reaction. Due to this, there is a discrepancy between the PVP content in the original composition and in the copolymer formulation. The structure of the copolymers was characterized by such parameters as the efficiency of grafting PVP (*f*), the composition of the copolymer, i.e., the ratio of HEMA:PVP in the copolymer, as well as the molecular weight of chain fragments between crosslinking nodes (*M_C_*), density (*µ*) and degree (*ν*) of crosslinking of polymer network (Figure 3). It was found that with increasing PVP content in the original composition, *µ* and *ν* decrease, which occurs due to increase of *M_C_*. The reason for this effect is that PVP macromolecules play the role of aerating agent of the polymer network. In addition, the part of the PVP that does not participate in the grafting reaction during hydration is washed away, increasing the free volume in the copolymer structure. Therefore, it is of interest to study the content of PVP (C_PVP_, %) in the copolymer depending on the initial composition formulation HEMA: PVP. For this purpose, the efficiency of PVP grafting and the PVP content in the copolymer, relative to its initial content in the original composition were calculated. It was found that with increasing the amount of PVP content in the original composition, its content in the copolymer increases, but the efficiency of grafting PVP in the copolymer decreases (Figure 3).

**Figure 4:**
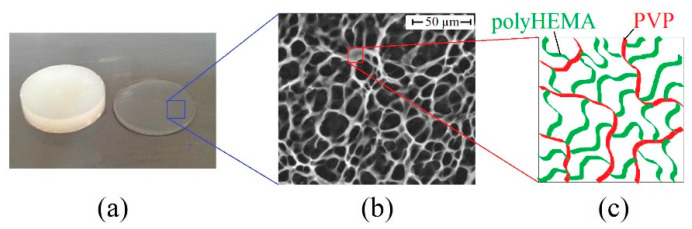
Swollen samples (**a**), SEM image of a porous hydrogel (**b**) and schematic image of the structure of pHEMA-gr-PVP copolymer (**c**) (Composition formulation, mass parts: HEMA:PVP = 80:20).

The presence of a porous structure of pHEMA-gr-PVP hydrogels is proved by photos of their macrostructure obtained by scanning electron microscope (SEM) (Figure 4). As shown, the obtained hydrogels represent a dispersed system consisting of pores, separated by a polymer.

The polymer from which the walls of the pores are formed plays the role of the framework, which is the basis of the hydrogel material. The nature of the pores in the hydrogel structure is influenced by the amount of unreacted washed out PVP. Such copolymers have a crosslinked structure, formed by HEMA blocks grafted on a PVP matrix (Figure 4c). In contact with the solvent, the hydrogel macromolecules are able to “move apart”, forming a free space. As shown, a hydrogel sample with a composition formulation of HEMA:PVP = 80:20 mass parts contains macropores with sizes in the range of 10 ÷ 30 μm, which in combination with the high complexing ability of the functional groups of both HEMA and PVP is a favorable condition for sorption and retention of low molecular weight substances [24].

### 3.2. The Properties of pHEMA-gr-PVP Copolymers

**Figure 5:**
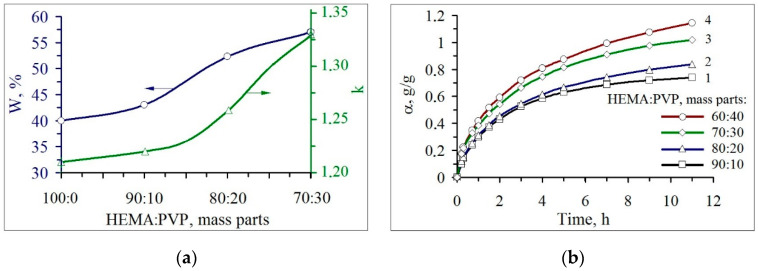
The effect of the initial composition formulation on the water content, the coefficient of swelling (**a**) and the kinetics of changes in the swelling degree α (**b**) of the copolymers.

The main performance properties of hydrogel materials are the ability to swell in solvents and diffusion-transfer properties. The paramount importance is the ability to swell in solvents, because this particular property determines the properties of the material, such as elasticity, biotolerance, and permeability for low molecular weight compounds. Copolymers of pHEMA-gr-PVP are characterized by a porous structure (Figure 4a) and contain hydrophilic groups: hydroxyl and carbonyl HEMA, as well as peptide PVP (Scheme 3). Precisely the number of such groups in the structure of the copolymer, as well as the porosity of the hydrogel determine its sorption capacity. The porous structure promotes rapid sorption of the solvent due to capillary forces, which causes swelling of the copolymers. The ability to swell was characterized by parameters such as boundary water absorption (W, %) and hydrogel swelling factor (k). With increasing PVP content in the composition, the water content and swelling coefficient of the synthesized copolymer increase (Figure 5a), which is explained by the hydrophilization of the network due to the introduction into its composition of chemically bound hydrophilic PVP chains, as well as the growth of intermacromolecular free volume in copolymer, due to outwashing the part of PVP which did not enter into reaction of grafting.

In addition, depending on the initial composition formulation, the structural parameters of the polymer network change: with increasing content of PVP, *M_C_* grows, i.e., the density of network crosslinking decreases (Figure 3).

Since the synthesized polymers are operated mainly in the swollen state, it is necessary to know the time of achievement of equilibrium swollen state by the material. Therefore, the kinetics of swelling of copolymers with different composition in water was studied (Figure 5b). It is established that the maximum swelling degree of samples with different composition formulation under normal conditions occurs after 24 h. As follows from the obtained dependences, the rate of swelling increases with increasing PVP content in the original composition.

**Figure 6:**
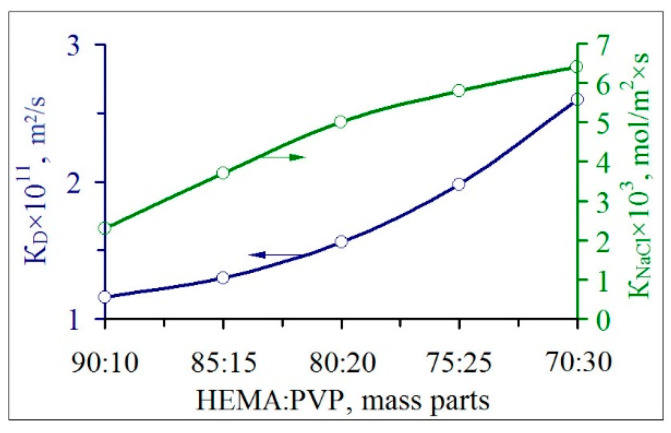
Dependence of diffusion coefficients (K_D_) and ion permeability (K_NaC_l) of hydrogels on the initial composition formulation.

The change of the crosslinking density of the polymer network and the efficiency of PVP grafting have a significant effect on the ion permeability of hydrogels as well. Permeability for low molecular weight compounds was investigated on the example of NaCl (Figure 6).

As shown, the ion permeability of hydrogels significantly depends on the initial composition formulation. With the increasing PVP content in the copolymer, the diffusion coefficient K_D_ and the ion permeability coefficient K_NaCl_ markedly increase as well. The growth of diffusion is promoted by an increase in the water content of the samples and an increase in their swelling.

Hydrogels based on pHEMA-gr-PVP copolymers are characterized by higher indexes of sorption capacity, ion permeability, oxygen permeability, compared with hydrogels based on polyHEMA. For example, it was found that the desorption of heparin in 24 h in aqueous medium is 40 times lower for polyHEMA, compared with pHEMA-gr-PVP copolymers [24], and oxygen permeability of hydrogels based on copolymers is 3–4 times higher compared to hydrogels based on polyHEMA [27].

During the creation of new products based on polymer hydrogels, mechanical strength is a fundamental problem, which in many cases limits the application fields of such materials. The evaluation of mechanical properties is essential in various biomedical applications viz ligament and tendon repair, wound dressing material, matrix for drug delivery, tissue engineering, and as cartilage replacement material [61].

Samples of pHEMA-gr-PVP copolymers obtained by block method are in a solid vitreous state and can be treated by cutting and sanding methods. Therefore, they must have sufficient hardness and heat resistance. At the same time, such materials are operated mainly in the swollen state, and therefore must have sufficient elasticity and tensile strength. Thus, the mechanical properties of the synthesized materials in dry and hydrated states were studied.

The strength of the copolymers in the dry state was characterized by surface hardness (F) and the ability of polymeric materials not to soften with increasing temperature was evaluated by heat resistance (using Vicat softening temperature (T_V_)).

**Figure 7:**
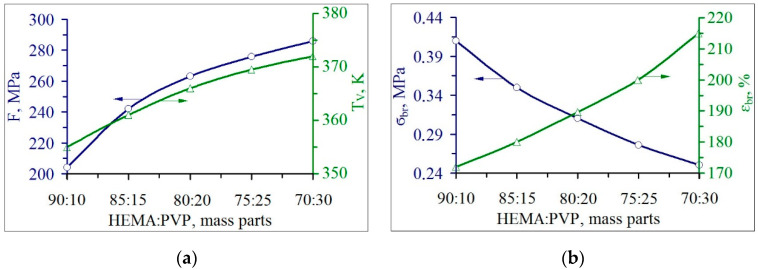
The effect of the initial composition formulation on the properties of block copolymers in the dry (**a**) and swollen state (**b**).

Block copolymers have a fairly high hardness, which increases with increasing PVP content (Figure 7a).

This can be explained by the introduction of macromolecular PVP chains into the polymer network. These chains contain bulky groups of cyclic structure, which effect the stiffness, i.e., reduce the ability of these chains to change conformation. The introduction of more rigid-chain PVP in the material contributes to the growth of T_V_. The growth of T_V_ also occurs due to the structuring of PVP as a result of HEMA grafting to it. The obtained research results of heat resistance of pHEMA-gr-PVP copolymers do not allow to draw a conclusion about the peculiarities of their destruction under the action of temperature. However, it is important to know if there is a need in mechanical and heat processes, when local overheating of the material is possible. Heat resistance of polymers is sufficiently characterized by derivatographic studies. The obtained results (Figure 2) short-term effect showed that the heat resistance of the material reaches 463 K, viz when processing this material into products, a short-term effect of temperature not higher than this value is allowed.

The strength of equilibrium swollen copolymers was characterized by tensile strength at break (σ_br_, Pa), and elasticity by elongation at break (ε_br_, %) (Figure 7b). The obtained data show that with increasing content in the PVP composition from 10 to 30 mass parts, tensile strength decreases significantly. Obviously, this is associated with a decrease in the number of macromolecules chains per unit volume of swollen polymer, which prevent the destruction, because increasing PVP in the original composition decreases the efficiency of its grafting (Figure 3). In addition, *M_C_* (Figure 3) and W (Figure 5a) increase with growth of PVP content in copolymer. However, for hydrogels, the increase in the water content and permeability is usually accompanied by a substantial degradation of their mechanical properties, and vice versa [39]. A significant increase (twice) of the elongation at break with increasing PVP content up to 30 wt.% is caused by the PVP effect on the composition and structure of the copolymer. As a result of the introduction of PVP macromolecules into the polymer, due to the grafting with polyHEMA, the network is formed with increased sizes of chain fragments between crosslinking nodes *M_C_* (Figure 3) which are characterized by an increased ability for conformational changes. Besides, the part of the outwashed PVP during hydration which did not take part in the grafting reaction increases the porosity of the polymer, which reduces the spatial barriers to conformational changes. In the case of tensile forces action, longer subchains between cross-linked nodes are able to straighten with a greater extent than short, which promote to increase the ε_br_.

## 4. Conclusions

The great advantage of the obtained materials is the possibility of their synthesis at a high rate, at room temperature in air without the use of additional stages of the technological process (vacuuming, purging the composition with inert gas). This result is provided by the presence of the stage of complexation between the participants of the process-monomer, PVP and Fe^2+^ ions, which leads to a decrease in the activation energy of polymerization. As a result of HEMA polymerization in the presence of PVP under the action of Fe^2+^ ions, grafted crosslinked copolymers with a set of unique properties are formed.

The interrelation of performance characteristics and structure of the synthesized copolymers is investigated. It is established that the directed change of the initial composition formulation is an effective way to regulate the structure (composition of the copolymer and the density of the network), and, accordingly, the physical–mechanical, transfer-diffusion and thermophysical properties of copolymers and their hydrogels. As the PVP content in the original composition increases, its grafting efficiency and the crosslinking density of the polymer network decrease. In this case, the hardness, heat resistance and sorption capacity of the copolymers in the dry state increase, as well as ion permeability and elasticity in the swollen state, but the tensile strength of the swollen hydrogels decreases.

The results of the work show that by changing the structure of the copolymer, it is possible to change its properties in the desired direction and obtain hydrogels with predetermined performance characteristics.

The use of iron (II) sulfate ensured the accomplishment of copolymerization of HEMA with PVP without the use of toxic initiation systems, which increases the possibility and prospects of using the obtained materials for the manufacture of products for medical purposes (contact lenses, implants, medical dressings, etc.).
